# Supplementary material for: Coordinated integrin activation by actin-dependent force during T-cell migration
Source: Nat Commun. 2016 Oct 10;7:13119. doi: 10.1038/ncomms13119 (PMC5062559; doi:10.1038/ncomms13119)
Supplement: Supplementary Information — Supplementary Figures 1-8 [file ncomms13119-s1.pdf]

a

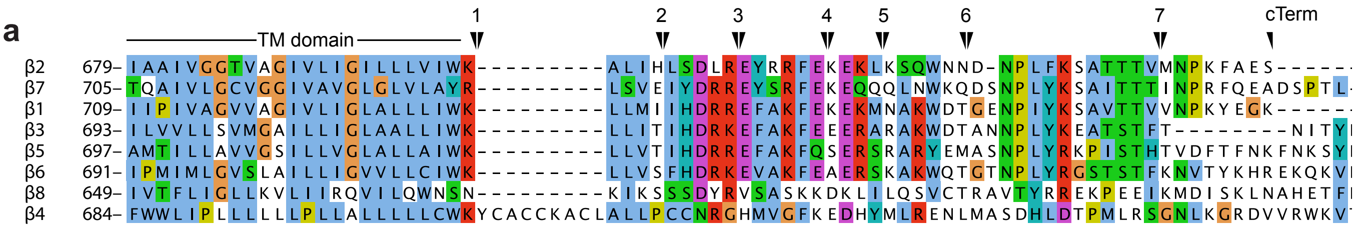

Supplementary Figure 1 Sequence alignment of integrin β-subunit cytoplasmic tails with indicated tension sensor insert positions in β2.

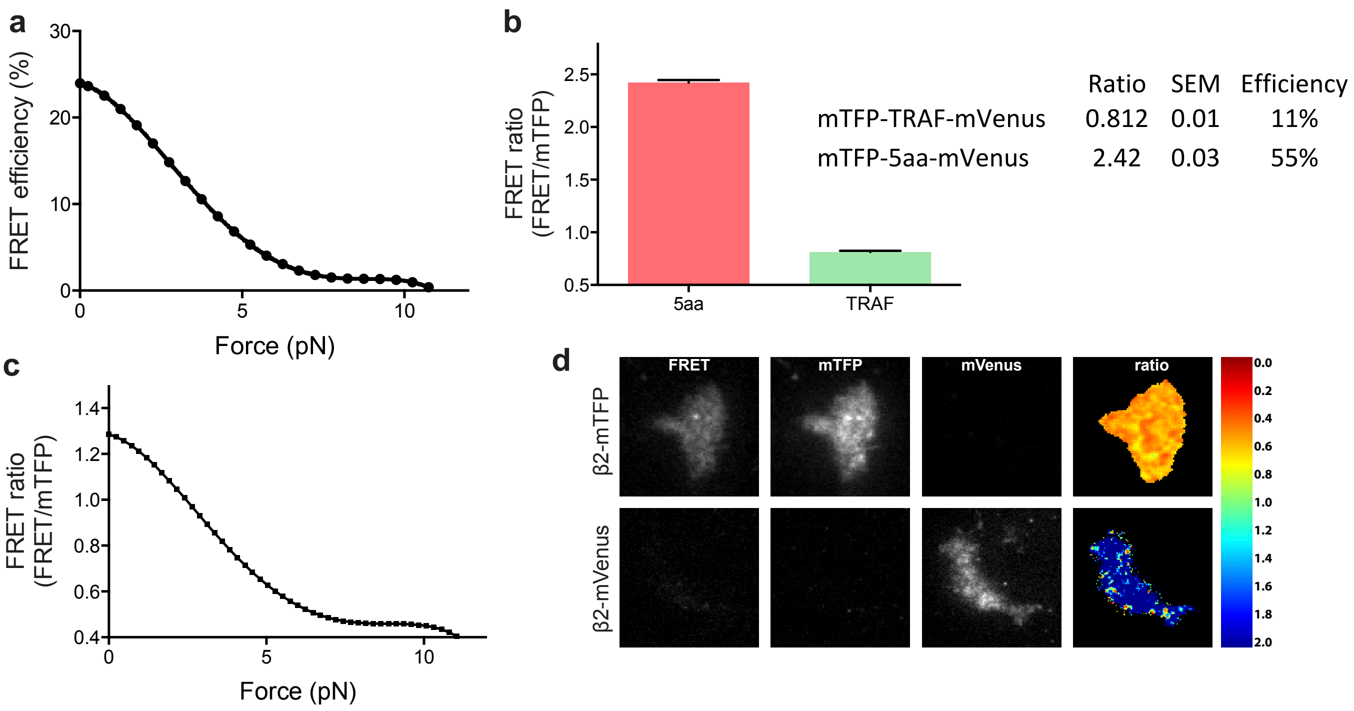

Supplementary Figure 2 Force estimation and FRET controls. (**a**) FRET efficiency and force relationship for the TS module (raw data from Grasshoff et al, Nature 2010). (**b**) Relationship of FRET ratio to FRET efficiency for the microscopy setup and image processing used in this work. 293T cells were transfected with mTFP-TRAF-mVenus or mTFP-5aa-mVenus and FRET data were collected, mean +/- SEM shown. These constructs have known FRET efficiences (11% and 55%). (**c**) FRET ratio and force relationship calculated from the data from B and C using linear interpolation. (**d**) Control images from Jurkat cells expressing either β2-mTFP or β2-mVenus and imaged in all FRET-related channels. From left to right: donor ex/acceptor em; donor ex/donor em; acceptor ex/acceptor em. Apparent FRET ratios (FRET/mTFP) are shown in the right panel.

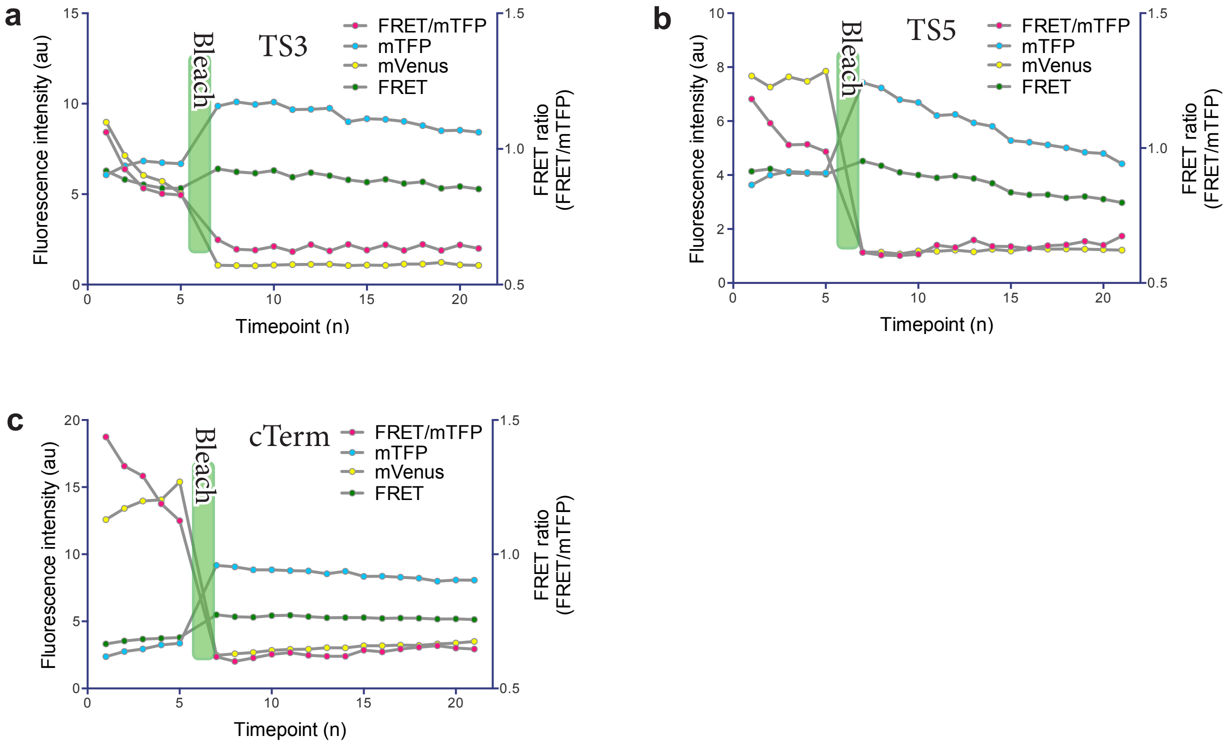

Supplementary Figure 3 Representative acceptor photobleaching curves for β2-TS3, -TS5, and -cTerm. (**a-c**) Five images were acquired before a 60s 515 nm bleaching at 100% laser power, and then all channels were acquired again for an additional 15 times. For all conditions the average FRET ratio after photobleaching was 0.65.

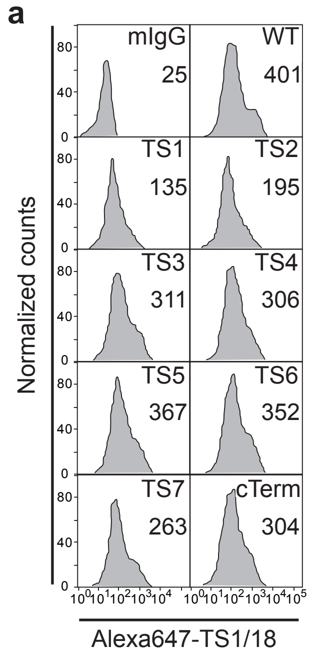

Supplementary Figure 4 Tension sensor expression data in 293T cells. (**a**) Representative histograms of data summarized in Fig. 2a. Mean fluorescence intenstities of Alexa647-labelled TS1/18 staining are indicated.

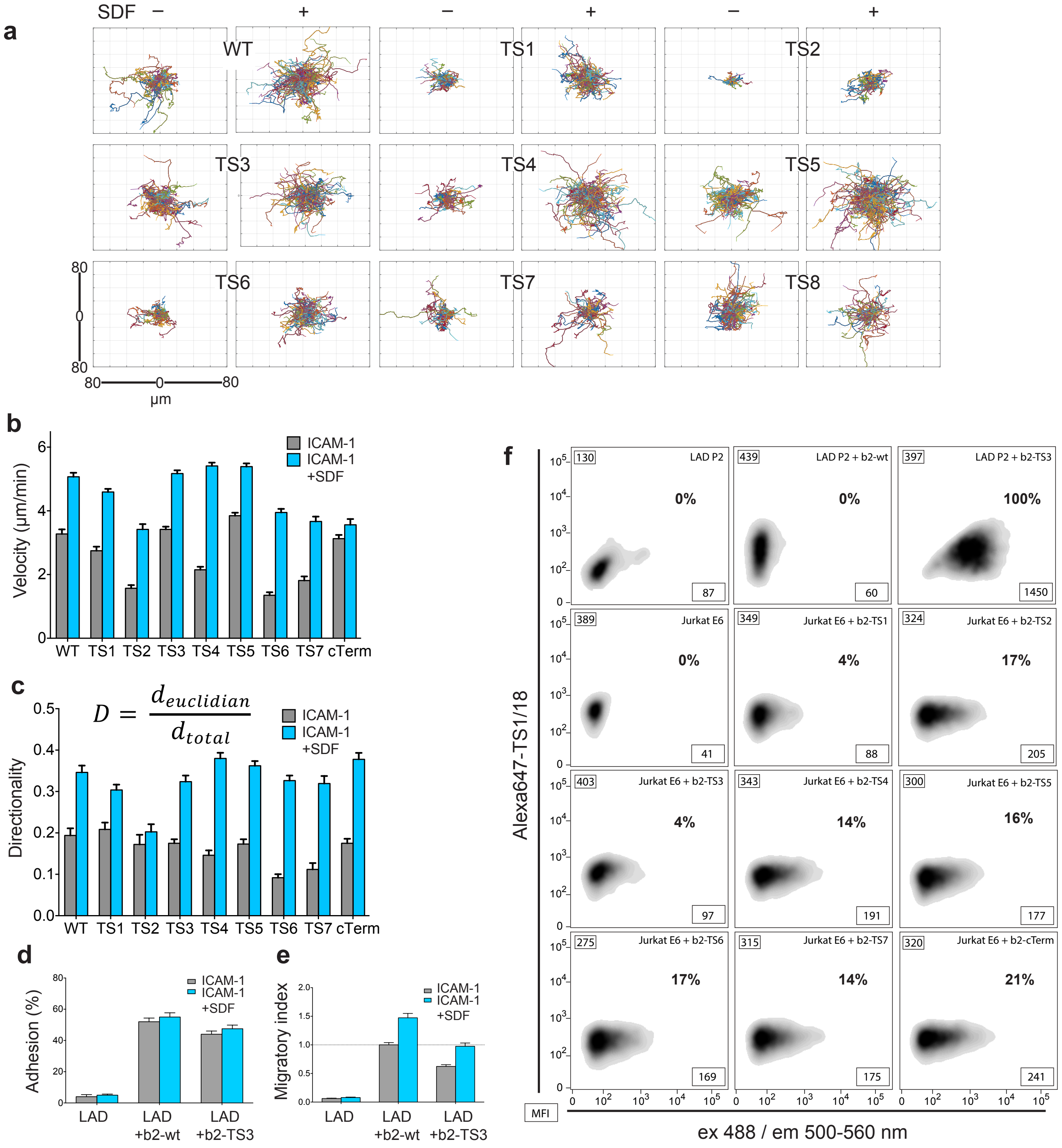

Supplementary Figure 5 Migration analysis and tension sensor expression data. (**a**) Representative cell tracks of cells used in Fig. 2. All tracks shifted so that starting position is at the origin. (**b, c**) Velocities (**b**) and directionality (**c**) of cell data used in Fig. 2B with mean +/- SEM. N is listed in Fig. 2b. Migration distance was measured each frame (30 s) to calculate velocity and total distance; the euclidian distance was between the initial and final position after 30 min of migration. (**d-e**) Adhered cells or migratory index of LAD cells transfected with either b2-wt or b2-TS3. Analysis was done in the same way as for Jurkat T cells. Mean +/- SEM of at least 100 cells from two experiments. (**f**) Expression of β2-integrin (Alexa 647–TS1/18) and tension sensor (green channel) in lentivirally transduced Jurkat T cells and LAD patient B cells. LAD cells with b2-TS3 were used for estimating the MFI ratio representing 100% LFA-1-TS integrins. Mean fluorescence intensity of all cells is shown in boxes. The Jurkat data was collected after a few weeks of migration experiments, and due to silencing effects the percentage of LFA-1-TS is likely slightly underestimated; this is particularly the case for Jurkat-TS3, which had been in culture for longer time than the others.

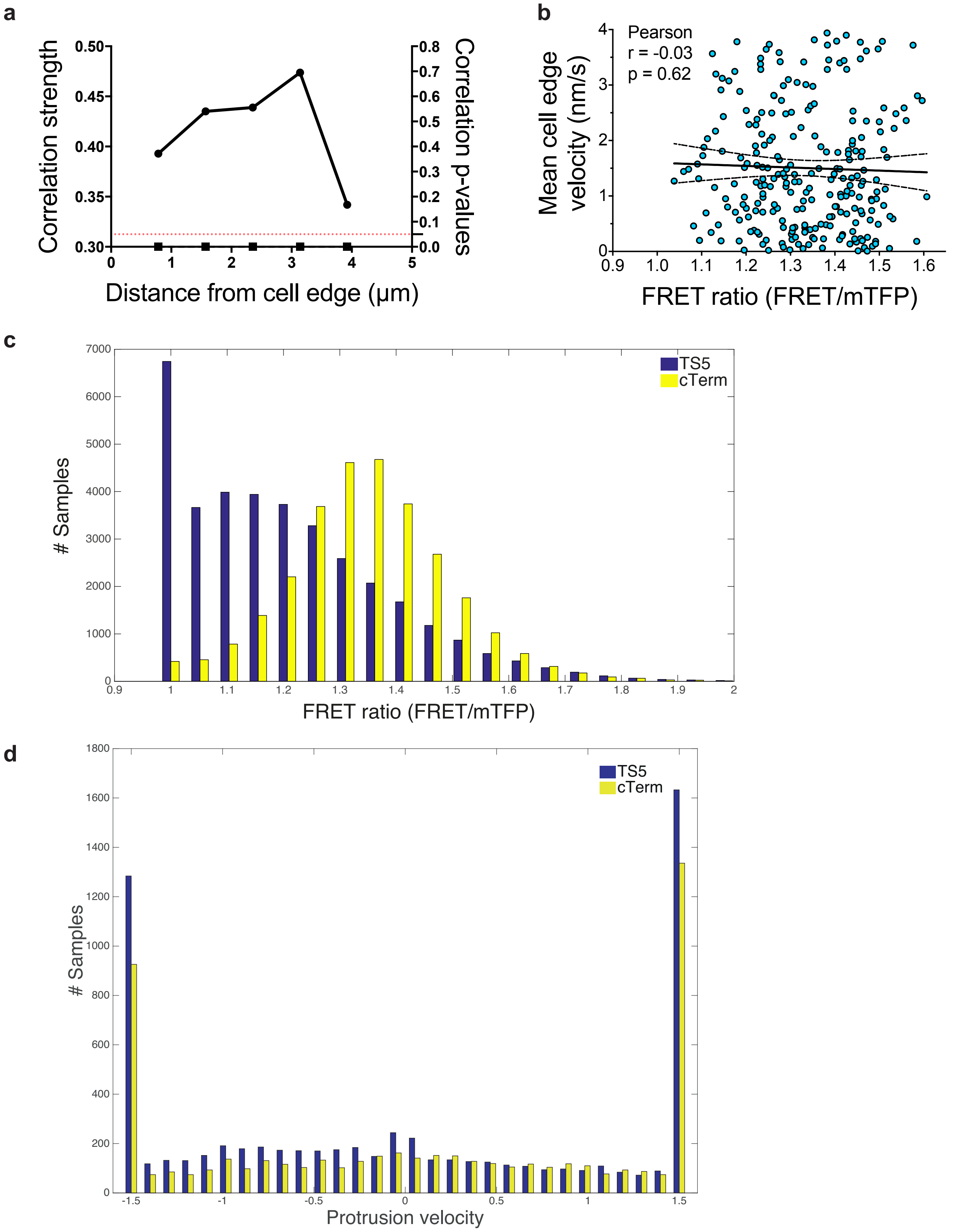

Supplementary Figure 6 Morphodynamics of b2-cTerm and comparison of FRET ratios and protrusion velocities for b2-Cterm and b2-TS5. (**a**) Correlation of FRET ratio vs protrusion localization for cTerm cells. Note that the sign is the opposite of that seen with TS5 in Fig. 7. (**b**) Distribution of FRET ratio vs protrusion velocity for cTerm cells with linear regression and confidence intervals overlaid. (**c**) FRET ratio distribution for TS5 and cTerm of the window data used for morphodynamics analysis. (**d**) Protrusion velocity distribution for TS5 and cTerm of the window data used for morphodynamics analysis.

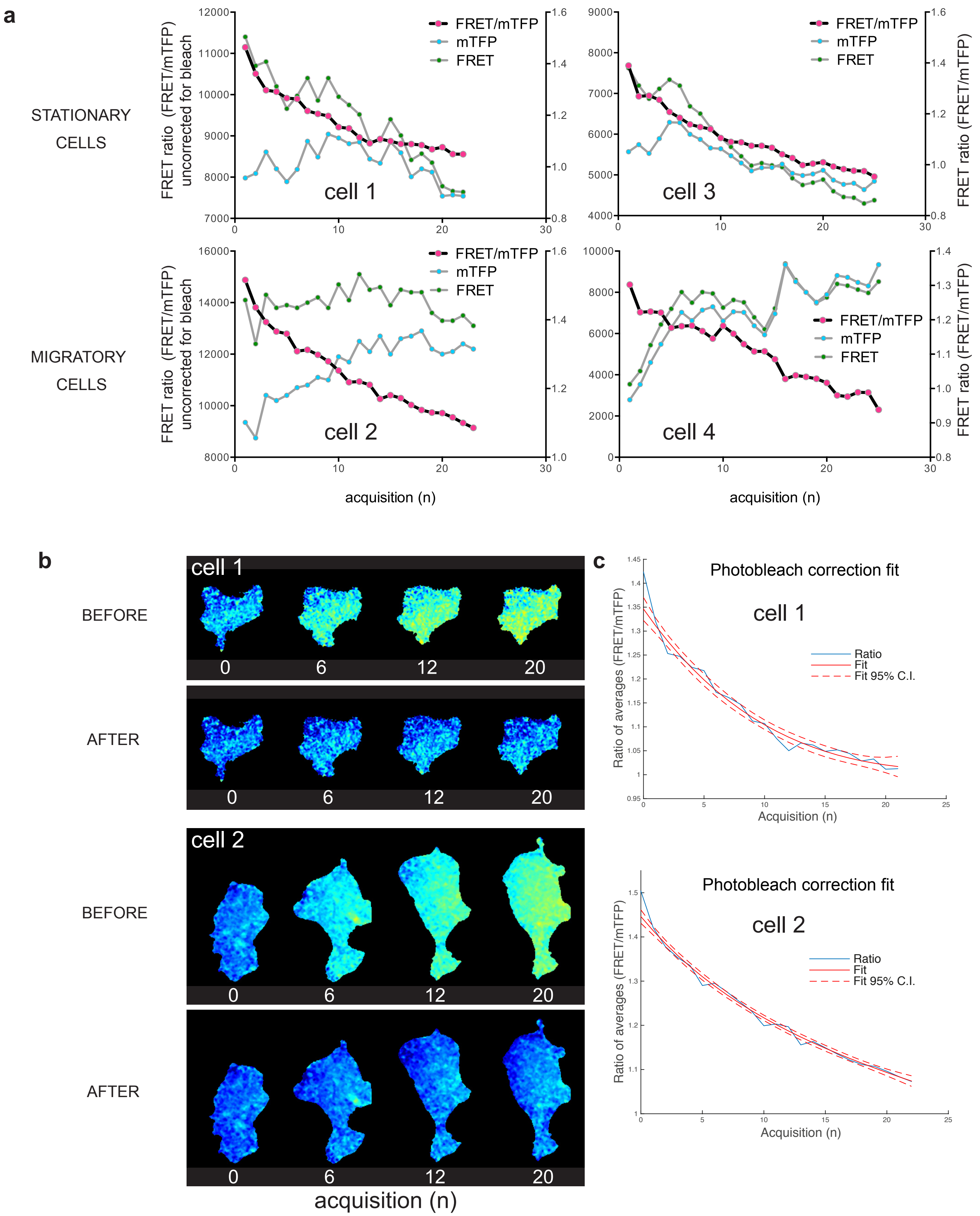

Supplementary Figure 7 Photobleaching in Jurkat T cells and correction of FRET data. (**a**) Representative examples of photobleaching in stationary and migratory β2-cTerm cells. Irrespective of how fluorescence intensities vary, the average FRET ratio decreases over time. (**b**) Example ratios from time series; numbers indicate acquisition number. (**c**) Single exponential fit photobleach correction examples using the ratio of average FRET and mTFP intensity across the cell for each frame. The ratio data are corrected by dividing the ratio with the fit values for each frame. See also Supplemental Movie 7.

a

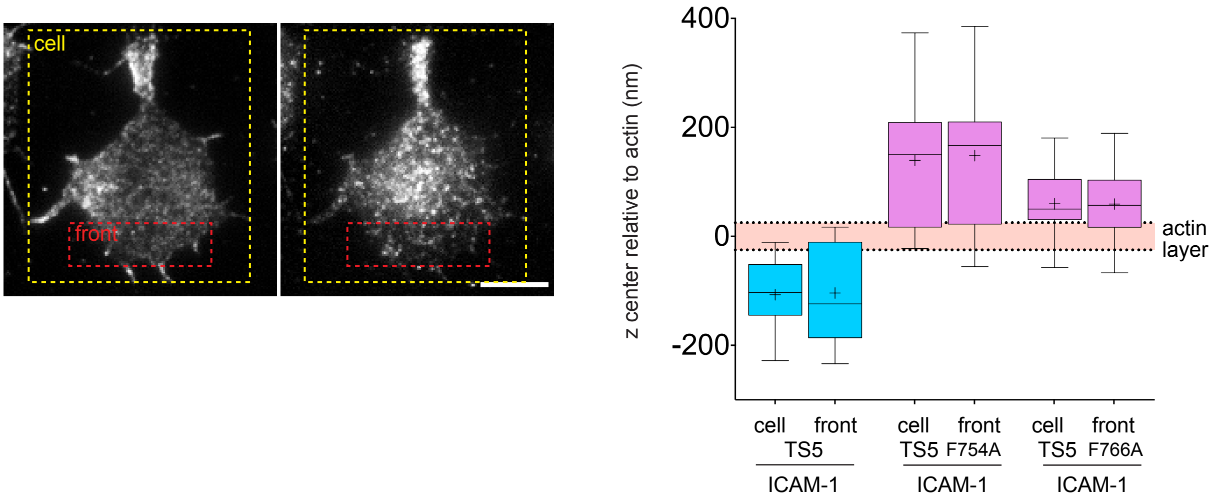

Supplementary Figure 8 – Localization-dependent control analysis for vertical localization of integrins. a. Representative images of focus plane in TIRF z-stacks (50 nm step) of Lifeact-mCherry and TS-mVenus with the indicated areas used for analysis. No significant differences were found between whole cell- and front-based analysis.
